# Supplementary material for: Community-level cohesion without cooperation
Source: eLife. 2016 Jun 16;5:e15747. doi: 10.7554/eLife.15747 (PMC4946899; doi:10.7554/eLife.15747)
Supplement: Supplementary file 1. — DOI: http://dx.doi.org/10.7554/eLife.15747.008 [file elife-15747-supp1.pdf]

Community-level cohesion without cooperation: Supplementary material

Mikhail Tikhonov

A. Relation to the model of MacArthur

The dynamics (3) can be written as

$$\frac{dn_{\vec\sigma}}{dt} = \frac{1}{\tau_0|\chi_{\vec\sigma}|} n_{\vec\sigma} \left(\sum_i \sigma_i H_i - \chi_{\vec\sigma} \right). \tag{S1}$$

where H_i denotes the "available resources". In the model considered in this work, $H_i = \frac{R_i}{T_i}$. MacArthur (1969) considered a model of species competing for renewing resources. In that model, the dynamics of organism populations were identical to (S1), but the availability of resources was given by $H_i = R_i(1 - T_i/r_i)$ (see equations (1)-(3) in MacArthur 1969), where the extra parameter r_i is the renewal rate (or the "intrinsic rate of natural increase").

The dynamics of the two models, therefore, differ only by the choice of the functional form relating population growth and the corresponding decrease of resource availability. The mapping between the notations of MacArthur 1969 ("MA") and those used here is provided in the table:

Notation for...	MA	Here
Species index	i	$\vec\sigma$
Species abundance	x_i	$n_{\vec\sigma}$
Resources a species can harvest	a_{ij}	σ_i
Resource carrying capacity	K_j	R_i
Minimal resource requirement	T_i	$\chi_{\vec\sigma}$
"Resource weight"	w_i	1
Resources $\mapsto$ biomass conversion factor	c_i	$(\tau_0\chi_{\vec\sigma})^{-1}$
Resource renewal rate	r_j	N/A

In the work of MacArthur, each species i was described by an arbitrary chosen vector of parameters a_{ij} (probability to encounter and consume resource j). The space of possibilities is unconstrained, and the types available to form a community are fixed by historical contingency; MacArthur then asks how many species can co-exist in this way. In the model considered here, a_{ij} are constrained to be 0 or 1. The setting is treated as an adaptive dynamics model where species are allowed to acquire or lose pathways, and the outcome of this co-evolution is investigated.

Reformulating community dynamics as an optimization problem was first done in MacArthur 1969; here, because of the difference in the way resource consumption is treated, the objective function being optimized is different, but the argument is similar. Consider the following objective function:

$$\tilde F = \sum_i R_i \ln T_i - \sum_{\vec\sigma} \chi_{\vec\sigma} n_{\vec\sigma}, \tag{S2}$$

defined for $\{n_{\vec\sigma} \geq 0\}$, and differing from the definition of Eq. (5) only by normalization and an additive constant.

Proposition 1: $\tilde F$ is bounded from above.

To see this, note the inequalities:

$$\sum_i T_i = \sum_{\vec\sigma} |\vec\sigma| n_{\vec\sigma} \leq N \sum_{\vec\sigma} n_{\vec\sigma}$$

and for $\alpha, \beta > 0$:

$$\alpha \ln x - \beta x \leq \alpha \ln \frac{\alpha}{e\beta}$$

Using these, and setting $\min_{\vec\sigma} \chi_{\vec\sigma} = \chi^* > 0$, one can write:

$$\tilde F \leq \sum_i R_i \ln T_i - \chi^* \sum_{\vec\sigma} n_{\vec\sigma} \leq \sum_i \left(R_i \ln T_i - \frac{\chi^*}{N} T_i \right) \leq \sum_i R_i \ln \frac{N R_i}{e\chi^*}$$

Proposition 2: $\tilde F$ is convex.

To see this, note that for any function $f(\vec n)$, the following two operations leave its convexity invariant (M is an arbitrary matrix):

1. adding a linear function of its arguments:

$$f(\vec n) \mapsto g(\vec n) = f(\vec n) + M\vec n;$$

2. performing a linear transformation of its arguments:

$$f(\vec n) \mapsto h(\vec n) = f(M\vec n).$$

Given these observations, convexity of $\tilde F$, and therefore also the convexity of F as defined in (5), directly follows from the convexity of the logarithm.

The main text demonstrated that $\tilde F$ is always increasing along the trajectories of the model. Thus, for any initial community state $\mathcal{C}$, ecological dynamics converge to the equilibrium corresponding to the unique maximum of $\tilde F$ on the domain $\{n_{\vec\sigma} \geq 0 \text{ for } \vec\sigma \in \Omega(\mathcal{C})\}$. Since $\tilde F$ is bounded and convex, the final equilibrium always exists and is unique and stable.

B. The role of the biomass assumption

When defining the dynamics (Eq. (3) in the main text), the biomass of species was taken to be equal to their cost. What role did this assumption play for the rest of the argument?

The main text describes an assay for evaluating the performance of a given species by placing a single individual into a "chemostat" with all resources supplied at equal abundance, and no other organisms present. There are two ways of defining the "intrinsic performance" of a species in this situation. For the purposes of this work, the most relevant measure is the largest population size this species can sustain; it is directly related to the threshold of substrate availability at which it can persist. However, an alternative measure could also be considered, namely the initial growth rate of the species in a pristine environment will all substrates untouched.

The "biomass=cost" assumption made in this work is intuitively convenient, since it makes these two measures of intrinsic performance coincide. In general, of course, one can imagine a fast-growing species that eventually yields to one that is slow-growing, but has a lower sustenance threshold of resource availability (*cf.* the classic argument by Tilman 1982). Allowing the biomass of species to be arbitrary would change this transient dynamics, decoupling the two measures of individual species' performance. However, the equilibrium states to which this dynamics converges would remain the same, as would the objective function F. None of the conclusions drawn in this work are sensitive to the choice of species' biomass, as long as the individual performance of a species is taken to mean its equilibrium abundance in a "pure culture" chemostat. The quantity defined in the main text as $f_{\vec{\sigma}}$ retains this interpretation for an arbitrary choice of the species' biomass.

C. Normalization of community fitness

The typical value of $\tilde{F}$ as defined in equation (S2) for a community close to equilibrium can be estimated as follows.

To estimate the first term, note that the cost per pathway of all organisms is close to χ_0, and therefore the overall expression T_i is approximately $T_i \approx R_i/\chi_0$.

The second term is the total cost of all organisms in the population $\sum_{\vec{\sigma}} n_{\vec{\sigma}} \chi_{\vec{\sigma}}$. At any equilibrium, it is equal to the total resource abundance $R_{\rm tot} \equiv \sum_i R_i$. This can be seen in two ways. One approach is to use the equilibria conditions to express the cost of all present organisms in terms of resources:

$$\forall \vec{\sigma} \in \Omega(\mathcal{C}):\, \chi_{\vec{\sigma}} = \sum_i \sigma_i \frac{R_i}{T_i}$$

Therefore,

$$\sum_{\vec{\sigma}} n_{\vec{\sigma}} \chi_{\vec{\sigma}} = \sum_i \left(\sum_{\vec{\sigma}} n_{\sigma} \sigma_i \right) \frac{R_i}{T_i} = \sum_i R_i.$$

Alternatively, this same equation can be derived from the condition of maximization of $\tilde{F}$, by setting $n_{\vec{\sigma}} \equiv M p_{\vec{\sigma}}$, and requiring $\frac{\partial \tilde{F}}{\partial M} = 0$. Both arguments lead to the same intuitive conclusion that at equilibrium, the total demand for a substrate must match the total supply.

Putting these observations together, the expectation for the value of $\tilde{F}$ at any equilibrium is therefore

$$\begin{aligned} \tilde{F} &= \sum_i R_i \ln T_i - \sum_{\vec{\sigma}} \chi_{\vec{\sigma}} n_{\vec{\sigma}} = \sum_i R_i \ln T_i - \sum_i R_i \\ &\approx \sum_i R_i \ln(R_i/\chi_0) - \sum_i R_i \equiv \tilde{F}_0 \end{aligned} \quad \text{(S3)}$$

When defining community fitness, it is natural to subtract this baseline value from $\tilde{F}$ as defined in (S2), and normalize by $R_{\rm tot}$:

$$F = \frac{\tilde{F} - \tilde{F}_0}{\sum_i R_i}.$$

This is the normalization chosen in equation (5) in the main text.

D. Sensitivity to the value of ϵ

Fig. 2B demonstrates that for small enough ϵ, the structure of the final equilibria does not significantly depend on this parameter. This can be intuitively understood as follows. Consider two resources A, B and organisms $\underline{A} = \{1,0\}$, $\underline{B} = \{0,1\}$, and $\underline{AB} = \{1,1\}$. If

$$\chi_{\underline{AB}} > \chi_{\underline{A}} + \chi_{\underline{B}}, \quad \text{(S4)}$$

it easily follows that the "generalist" organism $\underline{AB}$ will eventually be outcompeted by the two specialists $\underline{A}$ and $\underline{B}$. Conversely, if the opposite inequality holds, then $\underline{A}$ and $\underline{B}$ cannot stably coexist in the final equilibrium, since $\underline{AB}$ will always be able to invade, displacing one (or both) of them. In this way, in the metagenome partitioning model, community composition is shaped primarily by inequalities like (S4), which are invariant under changes in ϵ and depend only on the realization of the "noise" ξ.

E. The maximum number of coexisting types

The traditional question of how many types can coexist for a given set of parameters, although not at the focus of this work, is nevertheless instructive to address. A simple linear algebra argument demonstrates that in the model considered here, this maximum number is N: a stable coexistence is possible only for a number of types that is at most equal to the number of resources. This is because for a given set of K types, the K equilibria conditions $\Delta_{\vec{\sigma}} = 0$ can be seen as a linear mapping between the N-dimensional vector R_i/T_i and a K-dimensional vector of organism costs $\chi_{\vec{\sigma}}$. In the generic case (i.e. if no special symmetries exist in the cost structure), the existence of such a mapping requires $K \leq N$.

Symmetries in the cost structure can lead to degenerate equilibria circumventing this maximal coexistence

condition. Imagine, for example, that all organisms have the exact same cost per pathway χ_0. In this maximally degenerate case *any* combination of functional types can coexist, provided that $T_i = R_i/\chi_0$: no partitioning of metagenome is better than any other.

F. Numerical determination of community equilibrium

To determine the equilibrium state established through competition of a given set of K species, one could imagine choosing a random starting point with a non-vanishing abundance of all K competing species, and evolving it according to the dynamical equations for time $t \to \infty$. The Lyapunov function guarantees that such evolution would converge to an equilibrium state. However, if $K \gg N$ (for example, $K = 1023$ and $N = 10$ in Fig. 2A), such a procedure is highly memory-intensive and wasteful, since the final population is guaranteed to contain at most N types with non-zero abundance (see section "The maximum number of coexisting types").

Conveniently, verifying that a configuration is a final equilibrium is much easier than finding it: one only needs to check that the resource surplus $\Delta_{\vec{\sigma}}$ is zero for all competitors that survived and is negative for all those who went extinct. This verification is fast and is guaranteed to either confirm that the equilibrium state is correct, or provide a list of species that can invade it. Therefore, a simple heuristic procedure can construct the true equilibrium configuration through an iterated sequence of "guesses", whereby a subset of species is first equilibrated, and then updated by removing species that went extinct and adding those that can invade. This is the approach adopted here.

Specifically, calculations were performed in Matlab (Mathworks, Inc.). Availability of all resources was set to $R = 100$. The "initial guess" S_0 is constructed using the individual fitness criterion explained in the main text (low cost per pathway = high fitness): for each pathway i, the 10 most cost-efficient (lowest cost per pathway) functional types ($S_0^{(i)}$) that contained pathway i are determined; the union of these cost-efficient types, all taken at equal abundance of 1 unit, constitutes the "initial guess" $S_0 = \bigcup_i S_0^{(i)}$.

The following procedure is then iterated: community dynamics are simulated using MatLab's variable-order differential equation solver `ode15s` until the absolute magnitude of all time derivatives $\frac{dn_{\vec{\sigma}}}{dt}$ fall below threshold $10^{-4}\epsilon$. At this point, most of the very-low-abundance species still present in the community are in the process of exponential extinction. To ensure that all such low-abundance types are indeed going extinct, all types with abundance below 10^{-4} are removed from the population, the pruned community is re-equilibrated (to account for any tiny adjustments this removal might have caused), and the resulting state $\mathcal{C}^*$ is tested for being a non-invadable equilibrium. If any invaders are found, they are added to the community at abundance 1, and the simulation cycle is repeated. Otherwise (no species can invade), the configuration is accepted as being within the pre-determined numerical error of the true final equilibrium. This protocol ensures that in the community $\mathcal{C}^*$, the list of survivors is exact (because the invadability criterion is always checked for all competing species and is exact), and their abundance is within acceptable numerical error. The protocol always converged due to convexity of "community fitness" F.

Scripts performing calculations and reproducing Figs. 2–5 are available as Supplementary File 2.

G. Supplementary information for Figure 2B

Fig. 2B was generated as follows. For a given cost structure, 10 random subsets Ω_i of 100 types each were equilibrated to determine survivors S_i^*. The procedure was repeated for 10 random realizations of the cost structure at each ϵ, with ϵ ranging from 10^{-5} to 0.1. Thus for each value of ϵ, a total of 100 randomly constructed communities were evaluated. Fig. 2B shows the median performance rank of survivors S^* within the respective set of competitors, averaged over all 100 instances, where the median was either weighted (blue dashed line) or not weighted (red solid line) by abundance of the type at equilibrium.

H. Supplementary information for Figure 3

The trajectories displayed in Fig. 3 were simulated for time $T = 10^5$ starting from 10 random initial conditions whereby each of the 1023 types was set to an abundance value drawn out of a log-uniform distribution between 10^{-5} and 100. It is instructive to examine in detail the time traces of individual species shown in Fig. 3B for one community trajectory. Fig. S1A shows those same traces for the entire length of simulated time (of which Fig. 3B shows a part), and panel B shows the availability of each of 10 substrates at the matching time.

The randomly generated initial configuration is overpopulated; the total demand for the limiting resource $\sum_{\vec{\sigma}} n_{\vec{\sigma}}\chi_{\vec{\sigma}}$ vastly exceeds the total supply $\sum_i R_i$; equivalently, substrate availability is far below $\chi_0 = 1$, which is the value required to sustain an typical organism. As a result, population abundance crashes for all species, until substrate availability becomes close to 1 (Fig. S1B, left-hand side). Now the total supply and total demand are approximately matched ($\sum_{\vec{\sigma}} \chi_{\vec{\sigma}} n_{\vec{\sigma}} = \sum_i R_i$), and we enter the regime where optimizing community-level objective function is equivalent to minimizing $\sum_i \ln H_i$ (Fig. S1B, right-hand side). This is the regime discussed in the main text and shown in Fig. 3A, B, C.

The environment experienced by organisms at this stage is very dynamic. Collectively, substrates become progressively more depleted (as shown in Fig. 3C in the

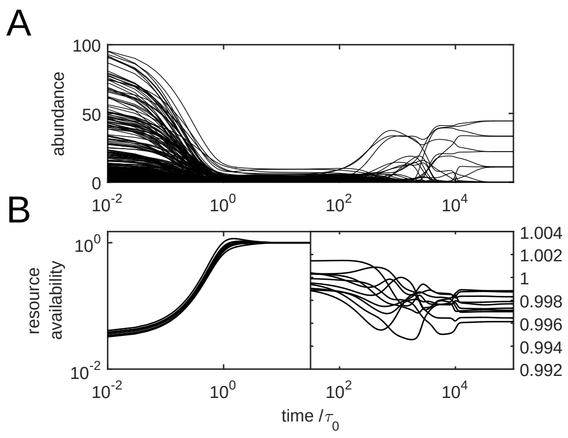

FIG. S1. **Convergence to equilibrium: a detailed view.** **A:** Same community trajectory of convergence to equilibrium as in Fig. 3B of the main text; entire simulation time is shown. **B:** Availability of individual substrates (the harvests H_i) during the convergence process shown in panel A. After the early stages of mass extinction in the overpopulated community, the availability of all substrates is brought close to 1, allowing species to sustain themselves. The scale of the Y axis is changed at this point, to show detail. Small adjustments of H_i at the later stages of convergence have a defining effect on the fate of individual species.

main text), however, the availability of individual substrates changes relative to each other (Fig. S1B, right-hand side). As a result, a species successfully growing at one point in time may end up going extinct at the final equilibrium. This highlights that the "cohesive" behavior of the community described in this work originates from this type of feedback between the organisms and the environment they shape for themselves.

I. Efficient resource depletion *vs.* efficient resource partitioning

Consider a three-species community

$$\mathcal{C}_0 = \{\underline{ABCDE}, \underline{FG}, \underline{HIJ}\}.$$

This community could be described as exhibiting efficient *resource partitioning* (each substrate is consumed by only one species). It is important therefore to highlight the difference between this kind of "efficiency" and the efficient resource depletion sought by the equilibrium community in the model described here.

Take the organism $\underline{ABCDE}$. There is a combinatorially large number of other species that could compete for the same substrates, some of which may have a lower cost per pathway, e.g. species $\underline{ABD}$. While it alone cannot fully displace $\underline{ABCDE}$, it will be able to invade, its lower cost causing the resources A, B and D to be depleted more efficiently. It is worth noting that the availability of C and E will go up, since their only consumer will reduce in abundance, but the overall efficiency as quantified by F move towards stronger substrate depletion. At the same time, the increase in availability of these two substrates means that the arrival of $\underline{ABD}$ is potentially facilitating another invasion.

The same goes for "cross-linkages" such as $\underline{DEF}$; the number of such species straddling the niches of current community members is again combinatorially large. Under the fully random cost model considered here, each of them is equally likely to possess a low cost by the luck of the draw, and it is therefore highly likely that at least some of them will be able to gain a foothold in the community. Thus the cost model considered here makes it extremely unlikely that the non-invadeable community will implement a perfect resource partitioning as exhibited by the community $\mathcal{C}_0$ (but see Sup. Ref. [5], where a more nuanced cost model allows different regimes to be explored).

J. Recombination

It is interesting to note that a formal equivalence between a certain class of community dynamics on one hand, and population genetics with recombination on the other hand, had previously been discussed in the literature (Akin 1979), but it was of a very different type. A curious mathematical accident makes the Fisher's equation for natural selection in a diploid population formally equivalent to the pure strategy dynamic in a certain class of evolutionary games (Taylor and Jonker, 1978). In that scenario, recombination is understood in the standard way, and the evolutionary dynamics proceed through regular binary (win/lose) competition governed by fixed values of fitness (game-theoretic payoff of a strategy). Here, in contrast, the observation is that the phenomenon of community coalescence can be seen as a generalization simultaneously of competition and of recombination.

[1] Akin E (1979) *The geometry of population genetics.* Lecture notes in biomathematics, Springer-Verlag, Berlin, New York, 205 pp.

[2] MacArthur R (1969). Species packing, and what interspecies competition minimizes. *Proc Natl Acad Sci U S A*, 64, p. 1369.

[3] Taylor P, Jonker L (1978) Evolutionarily stable strategies and game dynamics *Math. Biosci.* 40, p. 145–156.

[4] Tilman D (1892) *Resource competition and community structure.* Princeton University Press, Princeton, NJ. 296 pp.

[5] Tikhonov M (2015) Theoretical ecology without species. arXiv:1504.02550 (in review)
