# Supplementary figures and images for: Community-level cohesion without cooperation
Source: eLife. 2016 Jun 16;5:e15747. doi: 10.7554/eLife.15747 (PMC4946899; doi:10.7554/eLife.15747)

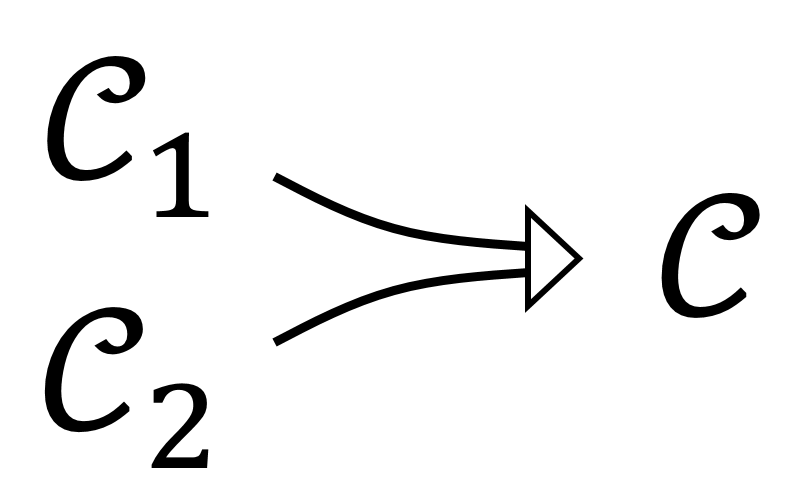

Supplement: Supplementary file 2. — DOI: http://dx.doi.org/10.7554/eLife.15747.009 [file elife-15747-supp2.zip › Cartoon_Fig4_H.bmp]
